# Metaphors are physical and abstract: ERPs to metaphorically modified nouns resemble ERPs to abstract language

**DOI:** 10.3389/fnhum.2015.00028

**Published:** 2015-02-10

**Authors:** Bálint Forgács, Megan D. Bardolph, Ben D. Amsel, Katherine A. DeLong, Marta Kutas

**Affiliations:** ^1^Kutas Cognitive Electrophysiology Lab, Department of Cognitive Science, University of CaliforniaSan Diego, CA, USA; ^2^Cognitive Development Center, Department of Cognitive Science, Central European UniversityBudapest, Hungary; ^3^Laboratoire Psychologie de la Perception, Université Paris DescartesParis, France

**Keywords:** metaphor, figurative language, ERPs, N400, concreteness effect, abstract-concrete, novel expressions

## Abstract

Metaphorical expressions very often involve words referring to physical entities and experiences. Yet, figures of speech such as metaphors are not intended to be understood literally, word-by-word. We used event-related brain potentials (ERPs) to determine whether metaphorical expressions are processed more like physical or more like abstract expressions. To this end, novel adjective-noun word pairs were presented visually in three conditions: (1) Physical, easy to experience with the senses (e.g., “printed schedule”); (2) Abstract, difficult to experience with the senses (e.g., “conditional schedule”); and (3) novel Metaphorical, expressions with a physical adjective, but a figurative meaning (e.g., “thin schedule”). We replicated the N400 lexical concreteness effect for concrete vs. abstract adjectives. In order to increase the sensitivity of the concreteness manipulation on the expressions, we divided each condition into high and low groups according to rated concreteness. Mirroring the adjective result, we observed a N400 concreteness effect at the noun for physical expressions with high concreteness ratings vs. abstract expressions with low concreteness ratings, even though the nouns *per se* did not differ in lexical concreteness. Paradoxically, the N400 to nouns in the metaphorical expressions was indistinguishable from that to nouns in the literal abstract expressions, but only for the more concrete subgroup of metaphors; the N400 to the less concrete subgroup of metaphors patterned with that to nouns in the literal concrete expressions. In sum, we not only find evidence for conceptual concreteness separable from lexical concreteness but also that the processing of metaphorical expressions is not driven strictly by either lexical or conceptual concreteness.

## Introduction

Metaphors are pervasive in everyday language, arguably being much more than mere rhetorical or poetic tools, possibly even serving as key instruments of linguistic change and innovation (Bréal, 1900). The high frequency of metaphors in natural language is taken by some to reflect the underlying metaphorical nature of the conceptual system. In their cognitive metaphor theory, Lakoff and Johnson (1980) propose that abstract target domains (e.g., *mind*) are structured and grounded via systematic mappings from concrete source domains (e.g., *containers*), thereby establishing conceptual metaphors (THE MIND IS A CONTAINER) which support everyday metaphorical expressions (e.g., “He couldn’t get the movie out of his head”). The term that refers to the source domain is also called the *vehicle*, the proposition that is stated about the *topic* term that in turn refers to the concept of the target domain. Even though the source domains are concrete, they are not intended for literal interpretation. For instance, the expressions “thick book” and “steamy book” are both noun phrases comprising an adjective evoking a physical property, followed by a noun. In the first case, the expression as a whole is understood literally, as an object with the physical property of thickness. In contrast, “steamy book” is not understood literally as a tome emitting steam but rather figuratively as a salacious romantic novel.

Embodied cognition (e.g., Lakoff and Johnson, 1999), however, argues the opposite, namely that metaphors are understood via the parallel co-activation of neural structures representing and/or processing physical properties (i.e., “steaminess” in the above example). Consistent with this hypothesis, Desai et al. (2011) found that metaphorical sentences involving physical motion (“The public grasped the idea”) were associated with fMRI activations of the left anterior inferior parietal lobe, a secondary sensorimotor area, just like sentences involving literal physical motion (“The girl grasped the flowers”). Moreover, since metaphors activated the left middle superior temporal sulcus similarly to abstract paraphrases (“The public understood the idea”), both an abstract and physical component are implicated. Less strict theories of embodied language processing (e.g., Binder and Desai, 2011) suggest that only novel expressions activate sensorimotor regions, and familiar expressions and/or familiar contexts rely on more abstract representations. This proposal resonates with language processing models such as the graded salience hypothesis (Giora, 1997, 2003) or the coarse semantic coding theory (Beeman, 1998; Jung-Beeman, 2005) that predict different processes for novel expressions (i.e., by the right hemisphere), regardless of figurativeness, and for conventional expressions (i.e., by the left hemisphere) as a result of their salient meaning and/or high degree of association. These potential differences between comprehension of conventional and novel metaphors fall outside the scope of the current inquiry, since we focus solely on relatively novel expressions.

Our aim in this report is to better understand the role that physical (or concrete, as we will also refer to them) properties of individual words (adjectives) and/or concepts (expressed by adjective-noun pairs) play in novel metaphor comprehension in real-time. To that end, we employ an online methodology that permits moment-by-moment examination of the metamorphosis from concrete, literal language into metaphorical, emergent concepts. Specifically, we recorded event-related brain potentials (ERPs)—a method enabling not just quantitative, but also qualitative, comparisons of the neural processing related to linguistic phenomena as they unfold in time.

ERP studies of metaphor processing are often centered on the N400 ERP component. The visual N400 (Kutas and Hillyard, 1980) is a negative-going centroparietally maximal ERP component peaking approximately 400 ms after stimulus onset, sensitive to the ease or difficulty of semantic memory access (for a review, see Kutas and Federmeier, 2011). Although the N400 is sensitive to a wide variety of factors that vary at the single-word level (e.g., concreteness, frequency, orthographic neighborhood size, repetition), the effects of top-down contextual information generally outweigh those of bottom-up information.

Several ERP studies have reported larger N400s to words appearing in metaphorical (e.g., “power is a strong *intoxicant*”) vs. literal (“whiskey is a strong *intoxicant*”) expressions (e.g., Pynte et al., 1996; Coulson and Van Petten, 2002, 2007; Tartter et al., 2002; Arzouan et al., 2007; Lai et al., 2009; Lai and Curran, 2013); overall, however, results have been inconsistent. Conventional metaphors (e.g., “broken heart”) have a fixed figurative meaning, and might be stored as lexical units (Jackendoff, 1997). Perhaps as a consequence they have been found to be processed faster and more accurately than novel metaphors (e.g., “rusty moves”), for which figurative meaning needs to be computed on-line (e.g., Faust and Mashal, 2007; Forgács et al., 2014). Investigations of novel metaphors, however, differ considerably in their details. Pynte et al. (1996), for example, modified the *topics* of conventional metaphors (“fighters” in “Those fighters are lions”), not the *vehicles* (“lions”) that carry the figurative meaning; in Tartter et al. (2002), sentence final words were not identical across conditions, leading to differences in frequency and cloze probability; (Coulson and Van Petten, 2002, 2007) controlled for the cloze probability of sentence final words, but not for the novelty and complexity of the expressions themselves (for further concerns with their stimuli see Lai et al., 2009).

Lai et al. (2009) carried out a well-controlled study, using a mixture of noun-, adjective- and verb-based metaphors in sentences of varying complexity. They showed that while conventional and novel metaphors both elicited larger amplitude negativities relative to literal sentences early in the N400 time window (320–440 ms), processing of conventional metaphors converged with that of the literal sentences, whereas novel metaphors continued to be treated more like anomalous sentences. They attributed the sustained negativity (between 440–560 ms) elicited by novel metaphors to semantic integration processes.

Figurative language also has been studied using semantically linked word pairs that constitute relatively minimal linguistic contexts. Arzouan et al. (2007) compared literal, conventional metaphoric, novel metaphoric, and unrelated two-word expressions by manipulating the first word while matching the second word on several psycholinguistic measures. They found that the N400 to the second word monotonically increased from literal, to conventional metaphorical, to novel metaphorical, to unrelated pairs. They also found differences in scalp topography and timing that suggest qualitative differences between the processing of conventional and novel metaphorical expressions; specifically they suggested that a late negative wave (between 550–880 ms) reflects secondary semantic integration, specific to novel metaphors. However, when novel metaphors are compared to conventional literal expressions or to sentences, novelty and figurativeness are confounded; hence the source of the effect is not clear. Comparing novel metaphors to conventional metaphors is not an optimal solution; firstly because it is, in essence, a manipulation of language conventionality; and secondly, and perhaps more importantly, there might be different processes involved in comprehending the two (cf., Bowdle and Gentner, 2005; Forgács et al., 2012). Together, these studies demonstrate, nevertheless, that metaphoricity influences real time language processing within the same time window (i.e., 200–900 ms post stimulus onset) as many other semantic factors (Kutas and Federmeier, 2011).

The N400 time window of the ERP is likewise sensitive to the concrete-abstract dimension of words, which might play a key role in the creation and comprehension of metaphors, which often involve mapping between an abstract (target) and a more concrete (source) concept. After controlling for potential confounding factors between concrete and abstract words, recent work shows behavioral processing advantages for abstract words (Kousta et al., 2011; Barber et al., 2013). ERP studies also indicate rapid differential processing of concrete and abstract words by about 300 ms post stimulus onset (e.g., Kounios and Holcomb, 1994; Holcomb et al., 1999; West and Holcomb, 2000; Lee and Federmeier, 2008; Barber et al., 2013; for a summary see Kutas et al., 2006). ERP concreteness effects are typically characterized as *greater negativity to concrete* *words* relative to abstract words starting within the N400 time window. These differences sometimes extend into a later time window (500–800 ms), where they typically exhibit more anterior, and more right lateralized, scalp topographies (e.g., West and Holcomb, 2000). These potentially separable electrophysiological constituents of the concreteness effect are consistent with Paivio (2007) dual coding theory, under which there are two semantic systems: a linguistic one that encodes both abstract and concrete words, and a non-verbal imagistic system that encodes only concrete words. On this theory, concrete words enjoy a processing advantage because they activate dual representations and tap into neural resources in both the linguistic and imagistic systems.

Concreteness effects have been observed in weakly constraining sentence contexts (e.g., West and Holcomb, 2000), as well as in single word contexts. Swaab et al. (2002) presented abstract and concrete words following (un)related prime words, and observed canonical N400 priming effects (larger amplitude N400s to words preceded by unrelated relative to related word primes) for both concrete and abstract words. However, they also found topographic differences between the abstract and concrete words, and an enhanced frontal negativity for concrete words, regardless of prime relatedness, consistent with structural and/or qualitative processing differences. It appears that in contrast to sentential contexts that eliminate topographic differences (Holcomb et al., 1999), single word contexts do not suffice to override qualitative ERP concreteness effects. Nonetheless, it seems that context also may have some bearing on the elicitation of concreteness effects.

Whereas minimal context ERP studies have typically manipulated concreteness by presenting different sets of concrete and abstract words that thus could differ on any number of other factors, Huang et al. (2010) cleverly relied on different adjective-same noun combinations to manipulate whether a given noun was modified in a concrete or abstract fashion. They conducted a divided visual field ERP study in which polysemous nouns (e.g., “book”) were presented in the left and right visual fields (LVF, RVF), modified either by abstract adjectives (“interesting”) or concrete adjectives (“thick”). Following flashes to the RVF (left hemisphere), concretely modified nouns (“thick book”) evoked reduced N400s (300–500 ms) relative to abstractly modified nouns (“interesting book”); this is the reverse of the canonical ERP concreteness effect. The authors suggested that concrete adjectives (which themselves evoked the canonical concreteness effect) established a more constraining context than abstract adjectives, and the resulting increased expectancy led to reduced N400s. Following LVF (right hemisphere) presentation, concrete (vs. abstract) expressions evoked a sustained negativity over frontal electrode sites only in a later 500–900 ms time window, consistent with previously reported qualitative processing dissociations, and therefore with some versions of the dual-coding theory. Based on the results of the two-word studies of Swaab et al. (2002) and Huang et al. (2010), the canonical (context-driven) N400 expectancy effect observed in published metaphor studies might be independent of the lexical concreteness effect seen in the same window, as the two effects seem to go in opposite directions.

To sum up, metaphorical expressions very often rely on physical expressions denoting concrete source domains to describe abstract target domains. Whereas figurative meaning clearly goes beyond the sum of its parts (i.e., the physical senses of constituent words), it is less clear to what extent (and when) the physical senses of constituent concrete words impact immediate processing of metaphorical expressions. Electrophysiological studies of metaphor processing generally show *smaller* amplitude N400s to literal relative to metaphorical expressions. In contrast, electrophysiological studies with centrally presented single words or expressions typically report a *greater* negativity within the N400 time window (and sometimes beyond) to more concrete relative to more abstract words. Against this background literature, we set out to assess whether metaphorical expressions created by combining physical adjectives that do not literally modify nouns (e.g., “sticky meeting”) are processed more like concrete or abstract adjective-noun expressions.

We adopted the word pair paradigm of Huang et al. (2010) in which different adjectives are combined with the same noun to rule out any potential lexical differences between target stimuli. Given that familiarity can mediate between concreteness and context effects (Levy-Drori and Henik, 2006), we limited our exploration to novel metaphorical adjective-noun word pairs, thereby ruling out conventional metaphors that might be stored in the lexicon, and thus invoke different processing. We compared and contrasted the following conditions, for which individual stimulus items were formed by combining three different adjectives with the same noun: (1) Abstract Literal (AL) expressions which were comprised of an abstract adjective + noun (e.g., “conditional schedule”); (2) Concrete Literal (CL) expressions which were comprised of a concrete adjective + noun (e.g., “printed schedule”); and (3) Metaphorical (MET) expressions which were comprised of a different concrete adjective + noun (e.g., “thin schedule”) that were likely to be interpreted metaphorically as they could not sensibly be interpreted literally (See Table 1 for additional representative stimuli).

**Table 1:** **Example stimuli**.

Metaphorical (MET) adjective	Concrete literal (CL) adjective	Abstract literal (AL) adjective	Noun
Fluffy	Nasal	Ineffective	Speech
Sticky	Loud	Constructive	Meeting
Stale	Scary	Comprehensive	Movie
Velvety	Hot	Protected	Lake
Magnetic	Slimy	Intelligent	Brain
Buzzing	Lively	Diligent	Receptionist
Gutted	Lush	Mystical	Forest
Fragile	Sloped	Unknown	Path
Wounded	Salty	Radioactive	Earth
Dripping	Soprano	Symbolic	Tone
Rusty	Painful	Improvised	Moves
Sparkling	Luxurious	Illegal	Party

At issue was whether processing of MET expressions would be driven (1) by the concrete (physical) nature of the adjective (e.g., “thin”)—in which case ERPs to the MET nouns would mimic those to the CL nouns; (2) by the non-literal abstract nature of the noun phrases—in which case ERPs to the MET nouns would mimic those to the AL nouns; or (3) by the non-literal, metaphorical nature of the noun phrase interpretation (e.g., “thin schedule”)—in which case ERPs to the MET nouns would differ from those to both the CL and AL nouns, eliciting the largest N400 and/or late negativity as in most ERP studies of novel metaphors.

We consider several potential outcomes in the N400 window (300–500 ms) of the adjective as well as the noun. We will first inspect the ERPs elicited by the adjectives to obtain a lexical concreteness effect baseline. We expect to see larger N400s to concrete adjectives (easily experienced with the senses) in both the CL and MET conditions compared to the abstract adjectives (not easily experienced with the senses) in the AL condition. If the concreteness of the adjective drives the processing and interpretation of the noun phrase, then we expect to see an N400 concreteness effect at the noun such that CL expression, but also the MET expressions, exhibit larger N400s than AL expressions (CL = MET > AL). Conversely, if it is the abstractness of the emergent concept to which the noun phrase refers rather than the abstractness of the adjective *per se* that drives processing and interpretation (such that the MET noun is processed as if it followed an abstract adjective) then the MET and AL nouns would elicit equivalently reduced N400 amplitudes (CL > MET = AL). If, however, the system concurrently distinguishes between emergent concreteness, and between abstract concepts that are literal vs. those that are metaphorical, then the N400 to metaphors may be even larger than the N400 for abstract expressions (MET > AL) due to increased processing demands of understanding a novel expression formed by an adjective referring to a physical trait that a noun cannot literally possess. This outcome would converge with the ERP metaphor literature, and with the differential sensitivity of the N400 to independent factors of concreteness and ease of processing/expectancy.

## Materials and methods

### Stimuli

To create the two-word expressions used in the ERP study, each of 212 nouns was combined with 3 different adjectives to form 636 novel word pairs. The nouns were polysemous in that metaphorical (MET), concrete literal (CL) or abstract literal (AL) expressions could result from modification by the different adjectives. Examples of the stimuli can be seen in Table 1.

The AL word pairs consisted of abstract adjectives modifying nouns to form expressions referring to abstract concepts. In the CL and MET conditions, adjectives were concrete, but in the MET condition adjectives modified nouns in a non-literal manner: 43% of the adjectives were shared across these two conditions. CL expressions referred to entities easily experienced by the senses, whereas AL and MET expressions referred to entities not easily experienced with the senses. Word pairs were designed to be meaningful but novel, with novelty controlled for by corpus measures. All word pairs appeared 4 times or less in the BNC and the probability of a noun following an adjective was less than 0.01. Semantic relatedness between constituents of expressions was low, as measured by Latent Semantic Analysis (LSA) (*M* = 0.11, *SD* = 0.11).

To ensure that stimuli were consistent with the definitions above, all word pairs were rated in an online norming study by 90 UCSD students not participating in the ERP study. Word pairs were rated along three dimensions (concreteness, literalness, and meaningfulness) on seven point Likert-scales: (1: not at all—7: completely). The three tasks assigned randomly to individual word pairs were: (1) “How easy is it to experience with the senses?”; (2) “How literal is it?”; and (3) “How meaningful is it?” We chose a literalness rating in order to avoid the explanation or definition of “metaphorical” and/or “figurative”, suspecting that it might be easier to determine whether something is meant literally than figuratively. Each participant saw every word pair but rated individual expressions along only one dimension. Across participants, all word pairs were rated for all dimensions. Pairs in which the CL expression was rated more abstract than the AL expression, or for which the MET was rated more literal than the CL or AL expressions, were excluded. Of the 212 normed items, the two rated least meaningful were discarded. Of the remaining 210 items, the half (105) rated most meaningful (and most literal and most metaphorical for the corresponding conditions) were used as stimuli, with the rest assigned to be fillers. Item norming statistics are summarized in Table 2. Using the same target nouns in each condition ensured that noun lexical factors were identically matched (i.e., no differences in terms of frequency, length or other psycholinguistic measure).

**Table 2:** **Means (standard deviations) of stimulus properties**.

	MET	CL	AL
Concreteness	3.46 (0.73)	4.89 (0.55)	3.85 (0.46)
Literalness	3.03 (0.58)	5.37 (0.72)	5.13 (0.7)
Meaningfulness	4.14 (0.74)	5.31 (0.64)	5.33 (0.56)
LSA	0.08 (0.09)	0.14 (0.13)	0.11 (0.09)

A one-way ANOVA revealed significant differences between conditions with respect to concreteness, *F*_(2,312)_ = 162.3, *p* < 0.001, $\eta_{p}^{2}$ = 0.51, literalness, *F*_(2,312)_ = 387.9, *p* < 0.001, $\eta_{p}^{2}$ = 0.71, and meaningfulness, *F*_(2,312)_ = 114, *p* < 0.001, $\eta_{p}^{2}$ = 0.42. Levene’s test of equality of error variances was significant for concreteness, *F*_(2,312)_ = 7.84, *p* < 0.001, and meaningfulness, *F*_(2,312)_ = 5.04, *p* < 0.01, and there was a strong trend for literalness, *F*_(2,312)_ = 3, *p* = 0.051. Therefore, the Tamhane *post hoc* test was used for pairwise comparisons. All conditions were significantly different from each other in concreteness (*p* < 0.001), and literalness (*p* < 0.05), while in meaningfulness AL and CL expressions were not significantly different, with only MET differing from the other conditions (*p* < 0.001, although all conditions were still above 4, the middle of the scale used).

Participants read each adjective-noun pair followed by a probe word that was either related or unrelated to the two-word phrase. Examples of stimuli and related probe words are shown in Table 3.

**Table 3:** **Example stimuli and related probe words**.

Condition	Adjective	Noun	Probe
MET	Fluffy	Speech	Exaggeration
CL	Loud	Meeting	Dispute
CL	Scary	Movie	Thrill
MET	Velvety	Lake	Beauty
AL	Unknown	Path	Hiking
CL	Salty	Earth	Coast
CL	Luxurious	Party	Tuxedo
MET	Rusty	Moves	Sports

### ERP participants

Forty-two UCSD volunteers (18 females) participated for course credit or were compensated at 7 h. Participants were right-handed, native English speakers with normal or corrected-to-normal vision, ranging from 18–29 years old (*M* = 21). Of the 42 participants, 7 were excluded from further analysis due to excessive eye blink or movement artifacts, which left a remaining 35 participants whose data we continued to examine.

### Procedure

The experiment was conducted according to human subject protocols approved by the University of California, San Diego Institutional Review Board. All participants provided their informed consent in writing before participating in the experiment. ERPs were recorded in a single session in a sound-attenuated, electrically shielded chamber. Participants sat one meter in front of a CRT monitor and read adjective-noun pairs followed by probe words. Participants used two hand-held buttons to indicate whether the probe word (e.g., “leader”) was related to the adjective-noun pair (e.g., “respected person”). Importantly, this technique encouraged participants to comprehend the novel metaphorical expressions in a figurative rather than a literal sense. Response hand was counterbalanced across participants and lists. Stimuli were centrally presented in white Arial 26 point font on a black background on a CRT monitor. Participants completed 6 blocks of 35 items each with short breaks between them. Each trial started with a blank screen (1000 ms), followed by a fixation cross “+” (1000 ms). The adjective appeared centrally for 200 ms, followed by a 300 ms blank screen, followed by the noun for 200 ms, followed by a 1500 ms blank screen, and finally a probe word appeared for 200 ms. After 800 ms following the probe onset, a question mark “?” was displayed until participants responded with a button press. A small red dot was presented centrally and slightly below the text throughout the trials, except during the question mark and the first 1000 ms blank screen; participants were instructed not to blink when it was present. Participants saw all 105 target nouns once, and each was paired with a single adjective once, resulting in 35 items from each condition, along with 105 filler expressions. Items were arranged in 5 different lists to avoid order effects. Each of the 5 lists was separated into 3 sublists so that each noun was paired with all 3 adjectives across participants.

### EEG recording

The electroencephalogram (EEG) was recorded from 26 electrodes arranged geodesically in an Electro-cap, each referenced online to an electrode over the left mastoid. Blinks and eye movements were monitored from electrodes placed on the outer canthi and under each eye, also referenced to the left mastoid. Electrode impedances were kept below 5 KΩ. The EEG was amplified with Grass amplifiers with a band pass of 0.01–100 Hz and was continuously digitized at a sampling rate of 250 samples/second.

### Data analysis

Trials contaminated by eye movements, excessive muscle activity, or amplifier blocking were rejected off-line before averaging: these trials (8.3% for MET, 10.6% for CL, and 10.1% for AL) were excluded from further analysis. Data were re-referenced off-line to the algebraic mean of the left and right mastoids and averaged for each experimental condition, time-locked to adjective onsets. ERPs were computed for epochs extending from 500 ms pre- to 1500 ms post-adjective onset, using a pre-stimulus baseline of 500 ms. Since we were interested in the processing of the two word adjective-noun expression, we baseline corrected only prior to the adjective, practically treating the two-word combination as one experimental unit.

ANOVAs were used to analyze mean amplitude ERPs over 6 medial central electrodes (MiCe, MiPa, RMCe, LMCe, LMFr, RMFr) where concreteness effects in the N400 time window are commonly observed: these were the same electrode sites over which adjective concreteness effects were assessed to determine inclusion in statistical analyses. Based on the literature, we analyzed concreteness effects in the following time windows: (1) an adjective N400 time window (300–500 ms post-adjective onset); and (2) a noun N400 time window (300–500 ms post-noun onset).

## Results

### Behavioral results

Overall response accuracy (*M* = 88%, SD = 6%) for button presses indicating whether or not the probe was related to the word pair suggested that the word pairs were read for comprehension.

### ERP results

**Figure 1:**
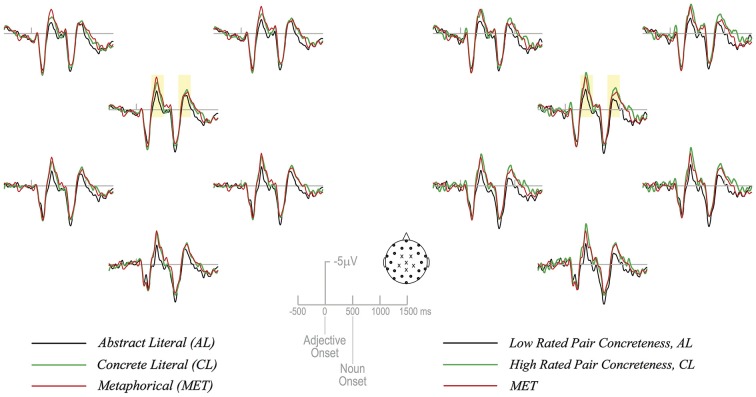
**The left and right panels show six central scalp electrodes from which ERP data were recorded and statistically analyzed (locations indicated with an X on the schematic array of 26 scalp electrodes)**. In the left half of the figure are grand average ERPs (*N* = 35) for the 3 experimental conditions. On the right half of the figure are grand average ERPs (*N* = 35) again for the MET condition, now contrasted with the lower half of the expression concreteness ratings (from the AL condition) and the top half of the expression concreteness ratings (from the CL condition). Target noun onset occurs 500 ms following adjective onset.

Average ERPs for all 35 participants are shown in Figure 1.

#### Adjective N400 (300–500 ms post-adjective onset)

An ANOVA with 3 levels of word type and 6 levels of electrode location revealed a main effect of word type, *F*_(2,68)_ = 10.65, *p* < 0.001, with CL and MET adjectives showing greater N400 mean amplitude (−1.78 µV and −2 µV, respectively) than AL adjectives (−0.67 µV). Planned pairwise comparisons indicated that the mean amplitudes for CL and AL adjectives and for MET and AL adjectives were significantly different (*p* < 0.001).

Our AL and CL conditions were based on adjective-noun pair concreteness ratings. However, to ensure that these labels also matched the concreteness of the adjectives alone, we obtained concreteness ratings for adjectives in the CL and AL conditions from Brysbaert et al. (2013). For 46 items not found in the database, concreteness ratings were collected from 7 UCSD undergrad students who did not participate in the ERP study. Adjectives were sorted into high and low concreteness conditions using a median split. An ANOVA with 2 levels of word type (high and low concreteness adjectives) and 6 levels of electrode location revealed a main effect of word type, *F*_(1,34)_ = 15.59, *p* < 0.001, with high concreteness adjectives eliciting a greater negativity (mean amplitude = −1.73 µV) than low concreteness adjectives (−0.62 µV). As these results were nearly identical to the results for our labeled conditions, we assume that the difference between AL and CL adjectives indeed reflects lexical concreteness.

#### Noun N400 (800–1000 ms post-adjective onset)

A concreteness effect in the expected direction is visible, with nouns in the CL condition eliciting a larger N400 than AL nouns. MET nouns appear to be patterning with CL nouns, also eliciting a larger N400 relative to AL nouns. However, an ANOVA with 3 levels of word type and 6 levels of electrode location showed no main effect of word type, *F*_(2,68)_ = 1.24, *p* = 0.3. In order to increase the sensitivity of the concreteness manipulation, we sorted the data based on paired concreteness ratings into the most concrete and least concrete items within conditions. The most concrete (top half of CL) and least concrete (bottom half of AL) items were compared to MET items in order obtain a clearer pattern of concreteness effects—if they were indeed present in the data.

An ANOVA with 3 levels of word type (MET, CL-high, and AL-low) and 6 levels of electrode location revealed a main effect of word type, *F*_(2,68)_ = 4.38, *p* < 0.05, with CL-high and MET nouns showing greater mean N400 amplitude (−1.39 µV and −1.17 µV, respectively) than AL-low nouns (mean amplitude = −0.29 µV) (Figure 1). Pairwise comparisons indicated that the difference between CL-high and AL-low nouns and MET and AL-low nouns was statistically significant (*p* < 0.05 for both comparisons). Thus the N400 pattern at the noun resembles that at the adjective.

**Figure 2:**
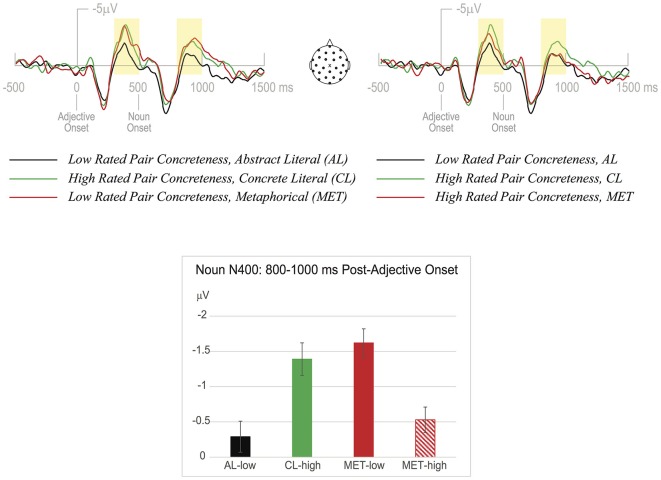
**The upper panels show ERP data at a representative midline central electrode (MiCe, aka Cz)**. The adjective N400 (300–500 ms) and noun N400 (800–1000 ms) time windows are indicated with shading. On the left, the ERPs associated with the low concreteness-rated MET expressions are contrasted with the lower half of the literal expression concreteness ratings (from AL) and the higher half of the literal expression concreteness ratings (from CL); on the right is the same comparison for the ERPs associated with the high concreteness-rated MET expressions. In the lower panel are N400 mean amplitudes (from the data in the upper panels) averaged across the 6 central channels indicated in Figure 1. Error bars indicate SEM.

Like the median split of the CL and AL conditions, we split MET items based on participant ratings of pair concreteness. These two conditions, MET-high and MET-low, were analyzed in order to better understand how metaphorical items may be processed on the basis of their rated concreteness. An ANOVA with 2 levels of word type (MET-high and MET-low) and 6 levels of electrode revealed a significant main effect of word type, *F*_(1,34)_ = 5.98, *p* < 0.05: the MET-low group was associated with a larger N400 mean amplitude (−1.62 µV) than the MET-high group (−0.53 µV). We next compared these two MET groups to the high and low CL ERPs (Figure 2).

First, the high-concreteness MET group was compared to the most abstract and most CL conditions described above. An ANOVA with 3 levels of word type (MET-high, CL-high, and AL-low) and 6 levels of electrode location revealed a main effect of word type, *F*_(2,68)_ = 3.18, *p* < 0.05, with MET-high nouns showing a reduced N400 mean amplitude (−0.53 µV) compared to CL-high nouns (−1.39 µV). Pairwise comparisons showed that the difference between MET-high and CL-high nouns was borderline significant (*p* = 0.08) and there was no statistical difference between MET-high and AL-low nouns (*p* = 0.58).

Second, the low-concreteness MET group was compared to the most abstract and most CL conditions described above. An ANOVA with 3 levels of word type (MET-low, CL-high, and AL-low) and 6 levels of electrode location revealed a main effect of word type, *F*_(2,28)_ = 4.10, *p* < 0.05, with MET-low nouns showing increased N400 mean amplitude (−1.62 µV) compared to AL-low nouns (−0.29 µV). Pairwise comparisons showed that the difference between MET-low and AL-low nouns was statistically significant (*p* < 0.05) and there was no statistical difference between MET-low and CL-high nouns (*p* = 0.66).

To ensure that the observed differences at the noun are not merely spillover from the adjectives, we conducted three ANOVAs as above in the adjective N400 time window (300–500 ms post-adjective onset). Comparing MET (−2.01 µV), CL-high (−2.35 µV), and AL-low (−0.8 µV) revealed a main effect of word type, *F*_(2,68)_ = 8.73, *p* < 0.001, with a significant difference between AL-low and both CL-high (*p* < 0.001) and MET (*p* < 0.01). The ANOVA including MET-high (−1.71 µV), CL-high and AL-low also showed a main effect of word type, *F*_(2,68)_ = 6.98, *p* < 0.01, where only MET-high was different compared to AL-low (*p* < 0.05). The ANOVA with MET-low (−2.32 µV), CL-high, and AL-low likewise revealed a main effect of word type, *F*_(2,68)_ = 7.64, *p* < 0.01, where only MET-low was different from AL-low (*p* < 0.01). In sum, while the pattern of N400 effects at the noun mimicked that at the adjective in the (high vs. low) literal conditions, this was not the case for the high vs. low MET conditions, which reversed their direction from adjective to noun.

## Discussion

In the current study we examined the real-time processing of novel metaphorical (“sticky meeting”), AL (“constructive meeting”), and CL (“loud meeting”) two-word (adjective-noun) expressions. We replicated the well-known N400 lexical concreteness effect on the initial adjectives of the two word expressions. A reliable concreteness effect also emerged for the nouns of the literal expressions when the most CL expressions were compared with the most AL expressions—despite the absence of any difference in rated lexical noun concreteness. We also found that on average the N400 to the metaphorical expressions patterned with that to the most CL expressions rather than with that to the most AL expressions, contrary to what we expected. Upon dividing the metaphorical expressions into more concrete vs. more abstract subgroups based on pair concreteness ratings we found that, paradoxically, the more abstract subgroup of metaphors were associated with a larger N400 than not only the most AL expressions but also the more concrete metaphor expressions.

The N400 concreteness effect on the prenominal adjectives resembles that reported for single words (Huang et al., 2010; Rabovsky et al., 2012; Amsel and Cree, 2013; Barber et al., 2013). This finding has been hypothesized to reflect activation of a richer network of semantic representations (or greater activation within a given network) during the processing of concrete vs. less concrete words. Within the literal expressions split by pairwise concreteness, target nouns elicited a prolonged negativity starting in the N400 time window that varied in amplitude with the rated concreteness of the expression. Huang et al. (2010) had showed that modifying a noun in a more concrete vs. more abstract manner can lead to a concreteness effect (e.g., for “book” in “interesting book” vs. “thick book”). Like Huang et al. (2010), we find that for literal (non-metaphorical) expressions, the concreteness of an adjective seems to determine the concreteness effect on a subsequent noun, at least at the extremes (the direction of their effect cannot be directly compared with ours as they employed visual half field presentation and a different task, among other differences). This manifestation of the concreteness effect at the noun is particularly striking given that the nouns themselves do not differ on this very measure (of concreteness).

Unlike some ERP studies (West and Holcomb, 2000; Huang et al., 2010), we find no evidence that our concreteness effects reflect imagery-related processes over and above the processes that routinely influence N400 amplitude. Specifically, our concreteness effects at neither the adjectives nor the nouns of literal expressions exhibited more frontal and/or right hemispheric distributions than the canonical N400 distribution to written words. As we already noted, our N400 concreteness effect at the adjective is consistent with a proposed richness of the activated conceptual representations in a lexico-semantic system (e.g., Holcomb et al., 1999; Barber et al., 2013). Following the same logic, our concreteness effect for the concretely vs. abstractly modified nouns could reflect the richness of the emergent higher-level conceptual representation—at least for the literal expressions. For example, reading “thick book” in order to determine its relation to an upcoming probe word could activate a richer network of features of the concrete concept BOOK than “interesting book”. This possibility does not necessarily implicate sensory/motor activations for the interpretation of the CL expressions, as it could just as well reflect greater activation within an amodal semantic system (Plaut and Shallice, 1993). Our results for the literal expressions extend the results of single word studies (e.g., Barber et al., 2013) and Paivio (2007) dual coding theory insofar as they demonstrate that concreteness need not be a strictly lexical property (i.e., pegged to single word meanings), but an emergent property of higher-level concepts as well.

For the metaphorical expressions, however, our N400 data pattern diverges from that of our literal expressions, as well as from Huang et al. (2010). When we compare the metaphor noun N400s to the noun N400s of the most abstract and most CL expressions, our data (at first glance) suggest that the concreteness effect at the noun is driven by the lexical concreteness of the adjective, as seems to be the case in the literal expressions and in Huang et al. (2010). To the extent that concreteness effects at the noun are merely an extension (spillover) of the ERP concreteness effect at the adjective, this pattern should remain unchanged for all metaphorical expressions. However, when we divide our metaphorical expressions by paired concreteness, the more concrete metaphors appeared to be processed (i.e., looked) more like AL expressions, and the more abstract metaphors looked more like CL expressions. In other words, the elicited negativity is reversed within the metaphorical expressions, with expressions rated as more abstract eliciting larger noun N400s than those rated as more concrete. If the negativity for metaphors observed in the N400 time window were a concreteness effect proper, high concreteness metaphors should have elicited a greater negativity than low concreteness metaphors. Yet the more concrete a metaphor was rated, the smaller the negativity it elicited. At a minimum, this pattern of results demonstrates that the processing of the nouns in the metaphorical expressions cannot be driven strictly by either lexical concreteness or higher-level emergent concreteness.

Of course, concreteness is only one of many factors known to influence the ERP, and in particular the N400. Less literal and more novel expressions have been shown to elicit larger N400s. Target words in novel metaphors usually elicit larger N400 amplitudes than target words in literal expressions (Coulson and Van Petten, 2002, 2007; Arzouan et al., 2007; Lai et al., 2009), and relative to conventional metaphors they elicit larger negativities slightly later as well, post-N400 (Arzouan et al., 2007; Lai et al., 2009). Contra this monotonic relationship, our high concreteness metaphorical expression condition did not elicit larger N400s than our AL condition, and strikingly, was* reduced* in comparison with CL expressions (see Figure 2). Even though their concreteness positively correlated with literalness (*r*_(105)_ = 0.59, *p* < 0.001) and the more abstract metaphorical expressions did elicit a larger N400 than the more AL expressions, they did not differ from the more CL expressions. If the increased negativity for metaphors in comparison with more AL expressions (Figure 1) were due to metaphoricity *per se*, it should have manifest for all metaphors, but it did not.

One reason why our results diverged in part from other investigations of metaphorical language may be that our expressions were matched on novelty across conditions, whereas in the aforementioned studies only the novel metaphors were unfamiliar. As a result, all three of our experimental conditions may have invoked some additional constructive or integrative processing (linked in previous reports to the post-N400, sustained negativity). On this possibility, our finding of equivalent N400s for more AL expressions and more concrete metaphorical expressions (despite a lexical concreteness difference at the adjectives) suggests that readers need not necessarily construct the literal (i.e., physical) interpretation of a novel metaphorical expression before understanding its figurative meaning. This interpretation is consistent with parallel models of metaphor comprehension (Glucksberg, 2003): the abstract, figurative meaning of metaphors might be readily and directly available, as also inferred by Blasko and Connine (1993). Our findings argue against other models of serial processing of metaphors as well. For example, Giora (1997, 2003) proposed that the comprehension of novel metaphors requires the rejection of a salient, literal meaning before arriving at a non-salient metaphorical meaning. If we assume that serial processing would result in non-identical ERP responses, the lack of differences between more concrete metaphors and more AL word pairs does not support the serial processing assumption (unless the latter have both a salient and a non-salient literal meaning).

Moreover, our results indicate that figurative meaning need not be directly derivative of the physical aspects of verbal expressions, but rather may at times emerge abstractly at least by the time window of the N400, a well-established marker of semantic analysis. Consequently, our data might pose a challenge to strong views of embodied cognition (e.g., Lakoff and Johnson, 1999). On a strong embodiment view, sensorimotor source domains (e.g., physical sensation of warmth) are activated in parallel with more abstract target domains, so as to provide structure and semantic content for understanding metaphorical expressions (as in “warm smile”). Gallese and Lakoff (2005) propose that “grasping an idea” involves some of the same motor activations as “grasping a banana”. In other words, during conceptual integration both conceptual domains should be active at the same time; if so, we expected to see this reflected in a canonical N400 concreteness effect at the noun. We did not. Among other possible interpretations, the apparent absence of a processing difference between more concrete metaphorical expressions and more AL expressions at the noun could be taken to mean that by the time some metaphorical meanings are constructed, physical aspects of the words might no longer be playing a tangible role in comprehension.

A cumulative conclusion thus far is that neither concreteness nor metaphoricity *per se*, can fully account for the processing differences among our novel literal and metaphorical expressions, at least in the N400 time window. We can speculate about what additional factor may be influencing our results. Among metaphorical expressions, rated pair concreteness correlates with meaningfulness, *r*_(105)_ = 0.53, *p* < 0.001 (a phenomenon observed also by Forgács et al., 2014). Thus, the greater N400 elicited by the less concrete metaphors could reflect the typical inverse relationship between context-driven expectancy and N400 amplitude, rather than processes specific either to lexical concreteness, or to figurative meaning. Perhaps the metaphorical expressions rated more concrete and more meaningful were more likely to increase semantic expectancies for the upcoming noun.

Our finding that more meaningful and more concrete metaphorical expressions seem to be processed like more AL expressions fits nicely with a newly emerging picture of metaphor comprehension. On this view, there is no empirical reason to assume that processing of metaphors invokes special processes that are not also required for comprehending literal language. Indeed, despite long held assumptions about the special role of the right hemisphere in figurative language, recent results suggest that it does not play a privileged role in metaphor comprehension after all (Rapp et al., 2004, 2007; Coulson and Van Petten, 2007; Bohrn et al., 2012; Forgács et al., 2012, 2014). Likewise, there is no support for the proposal that figurative meaning of novel metaphorical expressions proceeds only after attempts at (salient) literal meaning fail (Forgács et al., 2014).

Forgács (2014) has developed a novel theoretical framework for metaphor comprehension—Abstract Conceptual Substitution (ACS). According to this view, for an initial metaphorical interpretation it might suffice to substitute the vehicle term (“fluffy” in “fluffy speech”) with one of its abstract, non-physical properties, prior to any systematic mapping, or structural alignment, etc. This take on metaphor interpretation is closely related to that of Sperber and Wilson (2008), and the lexical pragmatic account of Wilson and Carston (2007), Carston (2010). They propose that metaphors are part of a continuum of loose language use (together with hyperbole and approximation, for example), which are understood via the generation of *ad hoc* concepts. For example, in the expression “fluffy speech” the concept FLUFFY is transformed into FLUFFY*, which is conceptually both broader and narrower (i.e., more general and more specific at the same time), in ways left as yet unspecified, than the original, encoded, lexical concept. Forgács specifies this broadening/narrowing in terms of the abstract-concrete dimension: FLUFFY* could broaden the lexical concept FLUFFY by activating more of its abstract properties (e.g., *superfluous, cushy*, etc.), but narrow the lexical concept by suppressing all of its concrete/physical properties (e.g., *physically protruding fluff, textile, texture*, etc.). This approach is similar to Glucksberg (2003) category assertion view, but does not rely on the creation of superordinate *ad hoc* categories or on the generation of *ad hoc* concepts. Instead, it might suffice to conceptually substitute the most relevant (i.e., contextually most activated) abstract property for the vehicle term (“fluffy”), creating “*superfluous, cushy* speech”. This is not merely a paraphrase, however, because expressing *superfluous* with “fluffy” brings along with it several cognitive consequences, such as deniability, negotiability, etc., much like indirect speech (cf. Pinker et al., 2008). The lack of a concreteness effect at least for the more meaningful, more concrete metaphorical expressions vs. the more AL expressions is consistent with this abstract substitution view in that the system seems to substitute abstract but not concrete properties for the vehicle term in our novel metaphorical expressions.

To sum up, our results suggest that the concreteness effect does not merely reflect the concreteness of individual words, but may also be sensitive to the concreteness of higher-level conceptual information. At least in the N400 time window, and seemingly only for more meaningful, more concrete adjectival metaphors, our findings suggest that metaphorical language may be processed and presumably understood in an abstract manner, despite the concrete nature of its constituent parts. In conclusion, it appears that comprehending certain metaphorical expressions created from physical concepts and words can be as *readily grasped*, and as *rapidly digested* as AL expressions, although not strictly driven by concreteness.

## Author contributions

Bálint Forgács conceived research; Bálint Forgács, Megan D. Bardolph, Ben D. Amsel, Katherine A. DeLong, and Marta Kutas designed research; Bálint Forgács, Megan D. Bardolph performed research; Bálint Forgács, Megan D. Bardolph, Ben D. Amsel, Katherine A. DeLong, and Marta Kutas analyzed data; Bálint Forgács, Megan D. Bardolph, Ben D. Amsel, Katherine A. DeLong, and Marta Kutas wrote the paper.

## Conflict of interest statement

The authors declare that the research was conducted in the absence of any commercial or financial relationships that could be construed as a potential conflict of interest.
